# Analyses of selected safety endpoints in phase 1 and late-phase clinical trials of anti-PD-1 and PD-L1 inhibitors: prediction of immune-related toxicities

**DOI:** 10.18632/oncotarget.18847

**Published:** 2017-06-29

**Authors:** Ricardo Costa, Rubens B. Costa, Sarah M. Talamantes, Irene Helenoswki, Benedito A. Carneiro, Young Kwang Chae, William J. Gradishar, Razelle Kurzrock, Francis J. Giles

**Affiliations:** ^1^ Division of Hematology Oncology, Northwestern University Feinberg School of Medicine, Chicago, Illinois, USA; ^2^ Northwestern University Feinberg School of Medicine, Chicago, Illinois, USA; ^3^ Northwestern University Department of Preventive Medicine, Chicago, Illinois, USA; ^4^ Center for Personalized Cancer Therapy, Moores Cancer Center, University of California at San Diego, La Jolla, California, USA

**Keywords:** anti-PD-1 antibodies, toxicity, clinical trial, solid tumors

## Abstract

**Purpose:**

Anti-PD1 and PD-L1 antibodies are associated with immune-related adverse effects (irAEs). This analysis aims to assess the discrepancies between frequencies of irAEs observed in phase 1 trials with those seen in late-phase trials and to evolve the field of drug development.

**Methods:**

PubMed search was conducted for articles published until December of 2016. Trials needed to have at least one of the study arms consisting of nivolumab, pembrolizumab or atezolizumab monotherapy. Trials were matched based on compound used and similarity of populations. All toxicities were reported as frequencies and percentages. *P*-values to assess differences between matches and non-matches of phase 1 and late-phase trials and between early and late-phase trials themselves were obtained via Fisher's exact test. Odds ratios were obtained via logistic regression.

**Results:**

Our search yielded 15 late-phase and 10 matching phase 1 trials; *n* = 4823 and *n* = 1650, respectively. The most common AEs seen in phase 1 trials were also observed in late-phase trials except for phase 1 trials (median *n* = 118) with < 118 patients (*P* = 0.048). Rash, pruritus, and diarrhea were the most frequently irAEs reported. Only colitis was more frequent in late-phase studies (*P* = 0.045).

**Conclusion:**

Toxicities of anti-PD-1 and PD-L1 observed in phase 1 trials and late-phase trials are similar. There is positive correlation between phase 1 trial sample size and concordance of toxicity frequencies seen in late-phase studies. In conclusion, current immunotherapy phase 1 trials are appropriate in assessing safety profile of anti-PD-1 and PD-L1 antibodies.

## INTRODUCTION

In the recent years numerous clinical trials have demonstrated the efficacy of anti-PD-1 and PD-L1 antibodies leading to FDA approval of nivolumab for patients with advanced melanoma, renal cell carcinoma (RCC), and non-small cell carcinoma (NSCLC) alongside pembrolizumab for treatment of advanced melanoma and non-small cell carcinoma NSCLC, and atezolizumab for urothelial carcinoma (UC) [1–6]. The efficacy of these drugs relies on enhancing anti-tumor immunity through the inhibition of negative regulatory signaling in T cells. The inhibition of immune checkpoint receptors disrupts immune tolerance resulting in enhanced immune activation in normal tissues leading to potentially significant toxicities [7]. Indeed, these agents have distinct toxicity profile compared to other therapies (i.e., chemotherapy and targeted therapies) characterized by immune-related adverse events (irAE) that are rare (absolute risk of all-grade irAE of ∼ 10%) but when present can result in life-threatening events [8–10]. irAEs affect a wide range of organs including endocrine organs, thyroid, adrenal and pituitary glands, skin, gastrointestinal tract, lung, kidney, liver, pancreas, and the nervous system [11].

As an inheritance from chemotherapy drug development methodology, conventional primary endpoints of phase 1 trials are: definition of maximum tolerated dose (MTD), recommended phase 2 dose (RP2D), and estimation of safety profile of new drugs to help guide later phase clinical trials (i.e., phase 2 and 3 trials) [12]. The underlying premise was of an existing relationship between dose-efficacy and dose-toxicity. Dose intensity was associated with anti-tumor efficacy [13, 14]. This paradigm is challenged by the observation of distinct late toxicities of newer targeted agents emerging from prolonged use of drugs under development as opposed to more traditional cytotoxic therapies associated with mostly acute toxicities [15]. Furthermore, checkpoint antibody (i.e., anti-PD1 and PD-L1 inhibitors) treatment has been designed to be administered over an extended period and traditional definition of MTD and dose limiting toxicity (DLT) may not allow for prediction of toxicities in later phase clinical trials. The goal of this analysis is to investigate the correlation between frequencies of adverse events observed in phase 1 trials of anti-PD-1 and PD-L1 monoclonal antibodies (i.e., pembrolizumab, nivolumab, and atezolizumab) with those seen in late-phase trials among patients with solid tumors with particular attention to irAEs.

## RESULTS

### Study inclusion and characteristics

**Figure 1:**
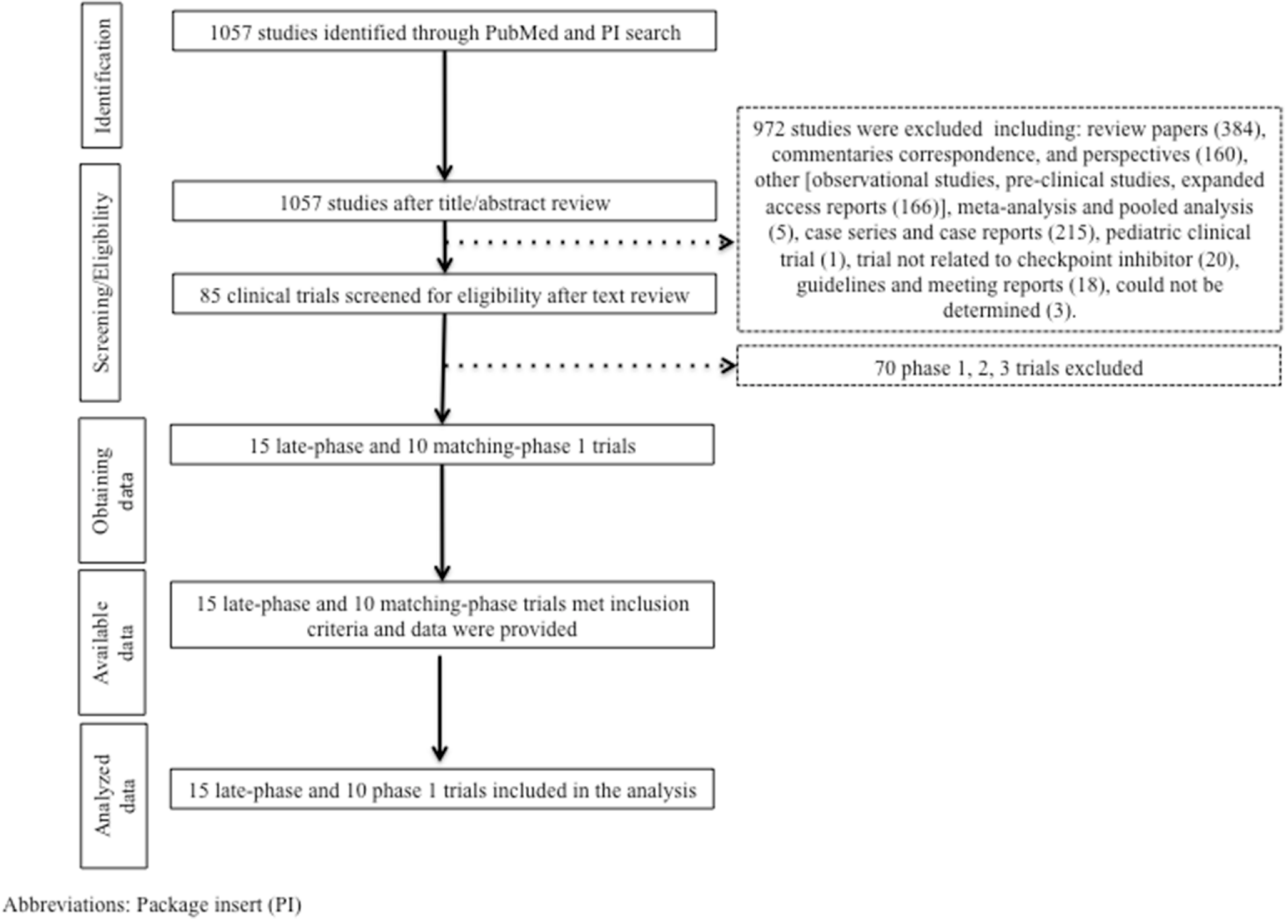
Study flow diagram Package insert (PI).

Initially our strategy yielded 1057 publications through our PubMed search. After screening of the study titles and abstracts 972 studies were excluded. After text review, 70 more studies were excluded for not meeting the inclusion criteria (Figure 1). Fifteen late-phase trials met the inclusion criteria and data were extracted. These studies comprised 10 phase 3, 4 phase 2 randomized studies, and one phase 2 nonrandomized trial. The latter was included into the analysis as it was the basis for approval of atezolizumab for the treatmentof UC (Supplementary Table 1). A total of 10 matching-phase 1 trials were included into our analysis. The number of dose levels ranged from 1 to 8. Three trials enrolled patients with different solid tumor histologies. Five trials studied nivolumab, 3 pembrolizumab, and 2 atezolizumab (Supplementary Table 2).

### Concordance between total number of AEs, grade 3/4, grade 5 AEs between phase 1 and late-phase trials

Death was a rare event among patients treated with checkpoint inhibitors. A total of 5 patients out of 1650 (0.03%) patients treated in phase 1 trial died as consequence of treatment-related AEs (all 5 were due to pneumonitis) compared to 12 out of 4823 (0.24%) in late-phase trials (i.e., encephalitis, myocardial infarction, sepsis, neutropenia, hypercalcemia one each; two due to pneumonia, four due to pneumonitis, and one due to unknown non-immune mediated causes; the odds of treatment-related deaths were similar comparing different clinical trial groups (OR = 1.2, 95% CI 0.6-2.4; *P* = 0.59). Grade 3 and 4 AEs were documented in 12% and 14% of the patients treated in phase 1 and late-phase studies, respectively (OR = 1.05, 95% CI 1.0-1.1; *P* = 0.052). Lastly, 69% of patients treated in the phase 1 trials group experienced an AE compared with 71% for the patients treated in the late-phase clinical trials (OR = 1.01, 95% CI 1.0-1.1; *P* = 0.04). These results suggest that phase 1 trials can reliably predict overall toxicities in late-phase studies.

### Concordance between irAEs in phase 1 and late-phase trials

The most commonly reported treatment-related irAEs reported in phase 1 trials were rash, pruritus, diarrhea, pneumonitis, and thyroid dysfunction (Table 3). Rash, pruritus, and diarrhea were the most common irAEs documented in both phase 1 and late-phase trials. Nine other immune-rated AEs occurred in similar frequencies in phase 1 and late-phase trials. At the trial level analysis, colitis was observed more frequently in late-phase trials compared to phase 1 trials (66.7% vs. 10%; OR=18; 95% CI 1.8-185; *P* = 0.01). Similarly at the patient-level analysis, all-grade colitis was reported at low frequencies in both phase 1 and late-phase studies but tended to be more frequent among the latter studies (0.12% vs. 0.85%; OR = 3.0, 95% CI 1.02-9.0; *P* = 0.045). There was higher frequency of hypophysitis, and adrenal insufficiency in late-phase trials but these differences did not reach statistical significance (i.e., 0.18% vs. 0.24% 0% vs. 0.12% in phase 1 and late-phase trials, respectively). All-grade pneumonitis and hypothyroidism were reported at high frequencies in both phase 1 and late-phase trials (70% vs. 86.7% and 70% vs. 73.3%, respectively) (Table 3). In summary, frequencies of irAEs were seen at similar rates in both phase 1 and late-phase studies expect for colitis.

### Concordance between most common AEs seen in phase 1 trials with AEs seen in late-phase trials

In the 10 phase 1 trials, fatigue, rash, pruritus and diarrhea were the most commonly reported all-grade AEs. In six out of ten matched-phase 1 trials the most common all-grade AEs were concordant with the most common AEs documented in the late-phase trials. Stratified analyses based on the following phase 1 clinical trial characteristics failed to show significant correlations between trial characteristic and the odds of concordance between frequency of most common AEs: (i) number of tumor histologies (multiple histology vs. monohistology), (ii) solid tumor type (melanoma vs. non-melanoma), (iii) checkpoint inhibitor type (nivolumab vs. pembrolizumab vs. atezolizumab), (iv) number of dose levels (≤ 3 vs. >3), and (v) number of patients evaluable for toxicity (Table 1). The number of patients evaluable for toxicity in each phase 1 trial ranged from 30-495, median = 118 patients. Five phase 1 trials enrolled more than 118 patients; all of those showed concordance of most common AEs with late-phase trials whereas only 1 trial with less than 118 participants showed concordance (*P* = 0.048).

**Table 1:** Concordance between the frequencies of most common treatment-related AEs in phase 1 trials and late-phase trials Question: Were the 4 most common AEs seen in phase 1 trials seen in late-phase trials?

Trial characteristics	*n* (early phase trials)	yes^$^	no^$^	*p*	Odds ratio (95% CI)
**Overall**	10	6	4		
**Multiple-histology phase 1**	10			0.13	
Yes	2	0 (0%)	2 (50%)		NA
No	8	6 (100%)	2 (50%)		
**Solid tumor histological type**	10			0.57	
Melanoma	4	3 (50%)	1 (25%)		3.0 (0.2, 48.0)
Non-melanoma	6	3 (50%)	3 (75%)		
**Treatment type**	10			0.99	
Nivolumab	6	4 (67%)	2 (50%)		
Pembrolizumab	2	1 (17%)	1 (25%)		0.5 (0.02, 12.9)
Atezolizumab	2	1 (17%)	1 (25%)		0.5 (0.02, 12.9)
**Number of dose levels in phase 1 trial**	10			0.99	
≤3	6	4 (67%)	2 (50%)		
3	4	2 (33%)	2 (50%)		0.05 (0.04, 6.7)
**Number of patients on phase 1 trials (median 118)**	10			0.048	
Above the median	5	5 (83%)	0 (0%)		NA
Below the median	5	1 (17%)	4 (100%)		

$ Defined as at least 50% (3/4) of the 4 most common AEs in phase 1 seen among most common AEs in later phase trials.

Data was reported as frequencies and percentages at the study level. *P*-values were obtained via Fisher's exact test. Odds ratios were obtained via logistic regression.

### Concordance between most common AEs seen in late-phase trials with AEs seen in phase 1 trials

In the 15 late-phase studies, fatigue, nausea, decreased appetite, and pruritus were the most commonly reported all-grade AEs. In 53% (8/15) late-phase trials the most common all-grade AEs were also observed at high frequencies in the matched phase-1 trials. By contrast in 47% (7/15) late-phase trials there was no concordance between the most common AEs and matched phase 1 trials. None of the late-phase trial characteristics showed significant correlation with the frequency of most common AEs seen in phase 1 trials: (i) clinical trial phase (phase 2 vs. phase 3), (ii) checkpoint inhibitor used (nivolumab vs. pembrolizumab vs. atezolizumab), (iii) line of therapy (1^st^ vs. 2^nd^ line or higher), (iv) tumor type (melanoma vs. non-melanoma), and (v) number of patients on late-phase trial (below or above the median).

## DISCUSSION

The value of phase 1 trials in predicting adverse events and accurately defining drug-related toxicity profile in the field of targeted therapies and chemotherapy drug development has been well documented [16]. In the recent years, anti-PD-1 and PD-L1 directed monoclonal antibodies gained unprecedented momentum in the field of cancer immunotherapy as illustrated by having three of those agents now approved for treatment of solid tumors (i.e., nivolumab, pembrolizumab, and atezolizumab). We conducted a systematic review to refine the understanding as to how well can phase 1 trials predict toxicities of immune checkpoint inhibitors in late-phase trials (phase 2 and 3). Fifteen late-phase studies (i.e., 14 randomized trials and 1 non-randomized phase 2 trial) met the inclusion criteria of this analysis, including 8 trials assessing the efficacy of nivolumab, 4 of pembrolizumab, and 3 of atezolizumab. These trials were matched with 10 phase 1 trials that supported advancement of these agents into corresponding late-phase trials. Matched phase 1 trials had 30 to 495 study subjects with median distribution of 118 participants. The elevated sample size observed among the 10 phase 1 trials analyzed represents presumed anticipated early efficacy signal of anti-PD1 and PDL-1 agents for the treatment of solid tumors leading to rapid transition to large expansion cohorts in these studies. Most matched phase 1 and late-phase trials showed similar frequencies of all-grade AEs and the most frequently AE reported in both phase 1 and late-phase studies besides fatigue affected the gastrointestinal tract and the skin (Supplement Table 1 and 2). This was not surprising as most common all-grade AEs related to checkpoint inhibitors have a peak frequency within less than 3 months of treatment initiation (i.e., diarrhea, rash, pruritus, etc.) [7]. By contrast, only in one out five studies with sample size below the median (*n* = 118) were the most common all-grade AEs observed among the most common toxicities in matched-phase 3 trials (*P* = 0.048), indicating the limitation of small phase 1 trials to detect AEs (Table 1). The large sample size of expansion cohorts observed in the phase 1 trials should also be noted. As expected, there was concordance between the frequencies of most common AEs observed among the 15 late-phase studies and the phase 1 studies in the majority of matched trials (Table 2). In our study, a total of 4823 and 1650 patients were evaluable for toxicity in all 15 late-phase and phase 1 studies, respectively. Indeed, all-grade rash, pruritus, diarrhea, pneumonitis and thyroid disturbances were the most frequent irAEs in both in early and late-phase studies (Table 3). Both at the trial and patient level analyses all-grade colitis was reported more frequently in late phase trials (*P* = 0.01 and *P* = 0.045, respectively) (Table 3). In general treatment with anti-PD-1 and PDL-1 inhibitors was well tolerated among patients with metastatic solid tumors. A total of 5 and 12 treatment-related deaths occurred in the phase 1 and late-phase studies, respectively (OR = 1.2, *P* = 0.59). Most of these deaths were secondary to lung toxicities (11 out of 17). Grade 3 and 4 AEs happened in 12% and 14% of the phase 1 and late-phase trials, respectively (OR = 1.05, *P* = 0.052), indicating similar and low frequencies of these events in early and late-phase trials. However notwithstanding similar frequencies of serious AEs leading to death the spectrum of grade 5 toxicities among patients treated with late-phase studies were much wider compared to phase 1 trials in which all 5 deaths were due to pneumonitis. Physicians and drug developers should be aware of and surveil for these potentially lethal toxicities. Nonetheless serious AEs leading to death were rare in both phase 1 and late-phase trials. Despite the overall large number of patients observed in most phase 1 studies, our findings are limited by the small number of immune checkpoint clinical trials published, which increases the chance of type 1 and 2 errors. In addition as this is a manuscript-based study we were limited in conducting further explorations of associations between study population characteristics and the predictability of toxicities in phase 1 trials, which would possible if we had access data at the patient level from each trial included. Furthermore, phase 1 trials herein reviewed were not matched with late-phase studies of equivalent dose and schedule of administration. The latter observation comes as a consequence of the fact that treatment regimens of anti-PD-1 and PD-L1 antibodies assessed in late-phase trials have been proposed based on pharmacokinetic and pharmacodynamic studies as opposed to conventional phase 1 endpoints (i.e., MTD, DLT, and recommended phase 2 dose level and schedule). Our results are important in that they validate the results of safety endpoints in phase 1 anti-PD-1 and PD-L1 treatments as a tool to predict toxicities in late-phase trials likely as a function of lager sample size of phase 1 clinical trials. This is important, as recent phase 1 checkpoint inhibitor trials have incorporated efficacy end points, which showed early treatment efficacy and led direct drug study in phase 3 trials [17, 18]. As newer therapies with distinct mechanisms of action continue to emerge the field of cancer drug development will need to continuously assess the appropriateness of phase 1 trials design in predicting toxicities in late-phase trials.

**Table 2:** Concordance between the frequencies of most common treatment-related AEs in late-phase trials and phase 1 trials Question: Were the 4 most common AEs seen in late-phase trials seen in phase 1 trials?

Trial characteristics	*n* (late-phase trials)	yes^β^	no^β^	*P*	Odds ratio (95% CI)
**Overall**	15	8	7		
**Late-phase trials**	15			0.61	
Phase 3	10	6 (75.0%)	4 (57.1%)		2.3 (0.3, 20.1)
Phase 2	5	2 (25.0%)	3 (42.9%)		
**Treatment type**	15			0.31	
Nivolumab	8	6 (75.0%)	2 (28.6%)		
Pembrolizumab	4	1 (12.5%)	3 (42.8%)		0.1 (0.007, 1.8)
Atezolizumab	3	1 (12.5%)	2 (28.6%)		0.2 (0.009, 3.0)
**Line of therapy**	15			0.99	
** 1^st^ line**	3	2 (25.0%)	1 (14.3%)		2.0 (0.1, 28.4)
** Not 1^st^ line**	12	6 (75.0%)	6 (85.7%)		
**Tumor type**	15			0.99	
Melanoma	5	3 (37.5%)	2 (28.6%)		1.5 (0.2, 13.2)
Non-melanoma	10	5 (62.5%)	5 (71.4%)		
**Median number of patients on late-phase trial (median 287)**	15			0.99	
Above the median	7	4 (57.1%)	3 (42.9%)		1.8 (0.2, 14.8)
Below the median	7	3 (42.9%)	4 (57.1%)		

β Defined as at least 50% (3/4) of the 4 most common AEs in late-phase trials seen among most common AEs in phase 1 trials.

Data was reported as frequencies and percentages at the study level. *P*-values were obtained via Fisher's exact test. Odds ratios were obtained via logistic regression.

**Table 3:** Concordance between potentially immune-related AEs events in phase 1 and late-phase trials Question: Were potentially immune-related AEs events seen in both phase and late-phase studies in similar frequencies?

Toxicity	Phase 1	Late-phase trial	*P*	Odds ratio (95%CI)
**Trial level analysis**				
	*n* = 10	*n* = 15		
Rash	10 (100%)	13 (87%)	0.50	NA
Pruritus	9 (90%)	12 (80%)	0.63	0.4 (0.04, 5.0)
Vitiligo	4 (40%)	5 (33%)	0.99	0.8 (0.1, 3.9)
Diarrhea	9 (90%)	15 (100%)	0.40	NA
Colitis	1 (10%)	11 (67%)	0.01	18 (1.8, 185)
Hypophysitis	2 (22%)	7 (47%)	0.23	3.5 (0.5, 22.3)
Adrenal insufficiency	0 (0%)	2 (13%)	0.50	NA
Hypothyroidism	7 (70%)	11 (73%)	0.99	1.2 (0.2, 6.9)
Hyperthyroidism	4 (40%)	9 (60%)	0.43	2.3 (0.4, 11.5)
Pneumonitis	7 (70%)	13 (87%)	0.36	2.8 (0.3, 20.8)
**Patient level analysis**				
	*n* = 1650	*n=* 4823		
Rash	223 (14%)	463 (10%)	0.36	1.0 (0.98, 1.1)
Pruritus	171 (10%)	503 (10%)	0.12	1.1 (0.99, 1.1)
Vitiligo	32 (2%)	134 (3%)	0.30	1.1 (0.95, 1.2)
Diarrhea	150 (9%)	554 (11%)	0.049	1.1 (1.0, 1.2)
Colitis	2 (0%)	41 (1%)	0.045	3.0 (1.02, 9.0)
Hypophysitis	3 (0%)	12 (0%)	0.19	2.1 (0.7, 6.5)
Adrenal insufficiency	0 (0%)	6 (0%)	NA	NA
Hypothyroidism	62 (4%)	240 (5%)	0.16	1.1 (0.98, 1.2)
Hyperthyroidism	17 (1%)	111 (2%)	0.15	1.2 (0.9, 1.5)
Pneumonitis	42 (3%)	114 (2%)	0.26	1.1 (0.9, 1.3)

Data was reported as frequencies and percentages at the study level. For trial-level analysis, P-values were obtained via Fisher's exact test.

For patient-level analysis, odds ratios were obtained via logistic regression. *P*-values and odds ratios were obtained via logistic regression.

## MATERIALS AND METHODS

### Search strategy

Package insert for currently approved anti-PD1 and PD-L1 antibodies were searched for references on late-phase approval trials (i.e., phase 2 and 3 trials) and phase 1 trials. For the PubMed search, the following keywords or corresponding Medical Subject Heading terms were used: “nivolumab” “pembrolizumab”, “atezolizumab”. The key word “ipilimumab” was also used to increase the sensitivity of our search, as phase 2 and 3 trials assessing the efficacy of ipilimumab combined or compared with anti-PD1/PD-L1 inhibitors exist. No filters or language limit was used to maximize search sensitivity. The database was searched for articles published until December 27^th^ 2016.

### Selection and matching of trails and data extraction

Late-phase trials were required to meet the following inclusion criteria: (i) at least one of the study arms consisting of nivolumab, pembrolizumab or atezolizumab monotherapy; (ii) solid tumor non-pediatric population, (iii) phase 2 trials were included only if they were used to support drug approval (Drug-approving phase 2 trials were allowed as our analysis aimed to assess the safety of current drug approval strategy of checkpoint inhibitors), (iv) late-phase trials not containing at least one of the antibodies under study as monotherapy were excluded. Phase 1 trials were required to meet the following inclusion criteria: (i) dose-finding study assessing toxicity of nivolumab, pembrolizumab or atezolizumab as monotherapy; (ii) solid tumor non-pediatric population. Finally matching between late-phase and phase 1 trials was performed according the following criteria: (i) compound with more than one matching phase 1 trial; larger matching phase 1 trial will be used for data analysis, (ii) the phase 1 clinical trial had similar patient population compared to the late-phase trial, (iii) when a late-phase trial could not be matched with a phase 1 single histology trial, the phase 1 multiple histology with larger number of patients with a given tumor histology was matched with the late-phase trial with corresponding tumor histology. For late-phase trials meeting inclusion criteria tumor histological type (melanoma vs. non-melanoma), and dose regimen were also documented. For phase 1 trials tumor histological type, number of dose levels, presence of dose-limiting toxicities were documented.

For all clinical trials, the first author's name, date of publication, study phase, and type of PD-1 or PD-L1 inhibitor were documented. The number of patients evaluable for toxicity and the number of adverse events (AEs) of phase 3 study arms containing the same PD-1 or PD-L1 inhibitor at different doses within the same clinical trial were summed for analysis. According to the National Cancer Institute Common Terminology Criteria Adverse Events (NCI CTCAE) version 4.0, the number of all grade, grade 3 and 4, grade 5, and potentially irAEs were extracted. The number of all grade potentially irAEs were collected according to study arm [rash, pruritus, vitiligo, diarrhea, colitis, hypopituitarism/hypophysitis, adrenal insufficiency, hypothyroidism, hyperthyroidism, hyperglycemia, non-infectious pneumonitis]. In light of the expected low number of irAEs, all grades of selected AEs were extracted. The four most common AEs observed in each phase 1 and late-phase study was also documented.

### Concordance of most common AEs in phase 1 and late-phase trials

The 4 most frequently observed all-grade AEs in both phase 1 and late-phase trials were documented. Matched late-phase and phase 1 trials were considered to have concordance frequencies of most common AEs if at least 50% (3 out of 4) of the 4 most common AEs in phase 1 seen among most common AEs in later phase trials.

### Statistical methods

Data were reported as frequencies and percentages at the study level. *P*-values to assess differences between matches and non-matches of phase 1 and late-phase trials and between early and late-phase trials themselves were obtained via Fisher's exact test. Odds ratios were obtained via logistic regression. *P*-values and odds ratios were obtained via logistic regression for the subject level analyses between early and late phase trials. Analyses were conducted in SAS v9.4.

## SUPPLEMENTARY TABLES




