# Supplementary material for: Analyses of selected safety endpoints in phase 1 and late-phase clinical trials of anti-PD-1 and PD-L1 inhibitors: prediction of immune-related toxicities
Source: Oncotarget. 2017 Jun 29;8(40):67782–9. doi: 10.18632/oncotarget.18847 (PMC5620211; doi:10.18632/oncotarget.18847)
Supplement: Supplementary file 1 [file oncotarget-08-67782-s001.pdf]

Analyses of selected safety endpoints in phase 1 and late-phase clinical trials of anti-PD-1 and PD-L1 inhibitors: prediction of immune-related toxicities

SUPPLEMENTARY MATERIALS

Supplementary Table 1: Late-phase studies included in the analysis

See Supplementary File 1

Supplementary Table 2: Matched-phase 1 clinical trials included in the analysis

PMID	Author year	Patient population	Number of patients evaluable for toxicity	Number of dose levels	Dose levels	MTD	RP2D	DLT	Four most common adverse events
25428503	Powels *et al.*	Bladder UC	68	1	Atezolizumab 15 mg/kg every 3 weeks	NA	NA	NA	Decreased appetite, fatigue, nausea, and pyrexia
22658127	Topolian *et al* 2.(2012)	Multiple histologies (solid tumors only)	296	5	Nivolumab 0.1, 0.3, 1, 3 and 10 mg/Kg every 2 weeks	Not reached	Not reported	Not reported	Fatigue, rash, diarrhea, and pruritus
25891174	Garon *et al.*	NSCLC	495****	3	Pembrolizumab 2 or 10 mg/Kg every 3 weeks or 10 mg/kg every 2 weeks	Not reported	Not reported	Not reported	Fatigue, pruritus, decreased appetite, and rash
25428504	Herbst *et al.* (2014).	Multiple histologies (solid tumors only)	277	8	Atezolizumab 0.01-20 mg/Kg every 3 weeks	Not reached	Nor reported	None observed	Fatigue, decreased appetite, nausea and pyrexia
25034862	Robert *et al.*(2014)	Melanoma	173****	2	Pembrolizumab 2 and 10 mg/Kg every 3 weeks	Not reported	Not reported	Not reported	Fatigue, pruritus, rash, and diarrhea
25897158	Gattinger *et al.*(2015)	NSCLC	129***	3	Nivolumab 1, 3 and 10 mg/Kg every 2 weeks	Not reached	Not reported	Not reported	Fatigue, decreased appetite, and diarrhea
25977344	Patnaik et al. (2015)	Multiple histologies (solid tumors only)	30	5	Pembrolizumab 1,3,10 mg/Kg every 2 weeks and 2,10 mg/kg every 3 weeks	Not reached	Not reported	None observed	Fatigue, nausea, pruritus, and decreased appetite
25800770	McDormett *et al.*(2015)	Advanced refractory RCC	34	2	Nivolumab 1 mg/Kg or 10 mg every 2 weeks	Not reached	Not reported	Not reported	Fatigue, rash, pruritus, and diarrhea
24145345	Weber *et al.* (2013)	Advanced melanoma 2nd line after ipilimumab	41**	NA	Nivolumab 3gmg/Kg every 2 weeks	NA	NA	Grade 3 rash	Fatigue, rash, pruritus, and diarrhea
24590637	Topalian *et al.*	Advanced melanoma (1 to 5th previous lines of therapy)	107	5	Nivolumab 1 mg-10 mg/Kg every 2 weeks	Not reached	Not reported	Not reported	Fatigue, rash, diarrhea, and pruritus

* There was a maintenance phase in this trial which is not described here due to space constraints.
Maximum tolerated dose (MTD), Not applicable (NA), non-small cell lung cancer (NSCLC), recommended phase 2 dose (RP2D), urothelial carcinoma (UC).
** Only cohort analyzed was the one treated with nivolumab monotherapy and human leukocyte antigen (HLA) unresctricted.
*** This is a dose expansion cohort with 3 dose levels from a dose-escalation phase 1 trial.
**** This is a randomized dose expansion cohort.
