# Supplementary material for: Analyses of selected safety endpoints in phase 1 and late-phase clinical trials of anti-PD-1 and PD-L1 inhibitors: prediction of immune-related toxicities
Source: Oncotarget. 2017 Jun 29;8(40):67782–9. doi: 10.18632/oncotarget.18847 (PMC5620211; doi:10.18632/oncotarget.18847)
Supplement: Supplementary file 2 [file oncotarget-08-67782-s002.docx]

| **Supplementary Table 1: Late-phase studies included in the analysis** | | | | | | | | | |
| --- | --- | --- | --- | --- | --- | --- | --- | --- | --- |
| PMID | First author (year) | Population  (line of therapy) | Number of patients evaluable for toxicity in anti-PD-1 treatment arms | Treatment  ARM 1 | Treatment  ARM 2 | Treatment  ARM 3 | Blinding | Pattern of randomization | Four most common adverse events |
| 25795410 | Weber *et al.* (2015) | Advanced melanoma  (2^nd^ or 3^rd^ line) | 268 | Nivolumab 3mg/kg  every 2 weeks | ICC  Dacarbazine 1000mg/m2 every 3 weeks  Or  carboplatin AUC of 6 with paclitaxel 175mg/mg every 3 weeks | NA | Open label | Randomization ratio 2:1, stratified by tumor PD-L1 status, *BRAF* status, and clinical benefit from previous. Permuted blocks (block size of six) within each stratum for randomization | Fatigue, pruritus, diarrhea, and nausea |
| 26406148 | Motzer *et al.*  (2015) | RCC  (2^nd^ or 3^rd^ line) | 406 | Nivolumab 3mg/Kg every 2 weeks | Everolimus 10mg daily | NA | Open label | Randomization ratio 1:1, block size of 4, with stratification by region (United States or Canada, Western Europe, and the rest of the world), MSKCC prognostic risk group, and the number of previous antiangiogenic therapy regimens (one or two) for advanced renal cell carcinoma. | Fatigue, nausea, pruritus, and diarrhea |
| 26412456 | Borghaei *et al.* (2015) | Non SCC NSCLC (2^nd^ line) | 287 | Nivolumab 3mg/Kg every 2 weeks | Docetaxel 75 mg/m2 every 3 weeks | NA | Open label | Randomization ratio 1:1, stratified by prior maintenance treatment (yes vs. no) and line of therapy (second line vs. third line) | Fatigue, nausea, decreased appetite, and asthenia |
| 26028407 | Brahmer *et al.*  (2015) | SCC NSCLC  (2^nd^ line) | 131 | Nivolumab 3mg/Kg every 2 weeks | Docetaxel 75 mg/m2 every 3 weeks | NA | Open label | Randomization ratio 1:1, stratified according to prior use of paclitaxel therapy (yes vs. no) and geographic region (United States or Canada vs. Europe vs. rest of the world [Argentina, Australia, Chile, Mexico, and Peru]) | Fatigue, decreased appetite, asthenia, nausea |
| 25399552 | Robert *et al.* (2015) | BRAF wild advanced melanoma  (1^st^ line) | 206 | Nivolumab 3mg/KG every 2 weeks plus Dacarbazine-matched placebo every 3 weeks | Dacarbazine 1000mg/m2 every 3 weeks plus nivolumab matched placebo every 2 weeks | NA | Double blind,  Placebo controlled | Randomization ratio 1:1, stratified by tumor PD-L1 status and metastasis stage (M0, M1a, or M1b vs. M1c, defined according to the tumor–node–metastasis system of the American Joint Committee on Cancer and the International Union against Cancer) | Fatigue, pruritus, nausea, and diarrhea |
| 26027431 | Larkin *et al.* (2015) | Advanced melanoma  (1^st^ line) | 313* | Nivolumab 3mg/Kg every 2 weeks | Nivolumab 1mg/Kg every 3 weeks plus Ipilimumab 3mg/Kg every 3 weeks for 4 dose followed by Nivolumab 3mg/Kg every 2 weeks* | Ipilimumab 3mg/Kg every 3 weeks | Double blind,  Placebo controlled | Randomization ratio 1:1:1, stratified by tumor PD-L1 status, *BRAF* mutation status, and American Joint Committee on Cancer metastasis stage (M0, M1a, or M1b vs. M1c) | Fatigue, rash, diarrhea, and pruritus |
| 26712084 | Herbst *et al*. (2015) | NSCLC  (2^nd^ line) | 682 | Pembrolizumab 2mg/Kg  every 3 weeks | Pembrolizumab 10mg/Kg  Every 3 weeks | Docetaxel 75mg/m2  Every 3 weeks | NO | Randomization ratio 1:1:1, stratified by ECOG PS (0 *vs* 1) and region (east Asia *vs* not east Asia), extent of PD-L1 expression. Treatment was allocated in blocks of six in each stratum | Decreased appetite, fatigue, nausea, rash |
| 26115796 | Ribas *et al.* (2015) | Ipilimumab resistant metastastic melanoma  (2^nd^ line) | 357 | Pembrolizumab 2mg/Kg  every 3 weeks | Pembrolizumab 10mg/Kg  every 3 weeks | ICC  (paclitaxel plus carboplatin, dacarbazine, or oral temozolomide) | Open label for chemo/ double blind for pembrolizumab dose | Randomization ratio 1:1:1, stratified by ECOG PS (0 vs. 1), lactate dehydrogenase concentration, and *BRAF* mutation status. Block randomization with a block size of six in each stratum | Fatigue, pruritus, nausea, decreased appetite |
| 25891173 | Robert *et al 2.* (2015) | Stage III or IV melanoma  (1^st^ or 2^nd^ line only) | 555 | Pembrolizumab 10mg/Kg  every 2 weeks | Pembrolizumab 10mg/Kg  every 3 weeks | Ipilimumab 3mg/Kg every 3 weeks | Open label | Randomization ratio 1:1:1, stratified by ECOG performance status (0 versus 1), line of therapy (first versus second), and PD-L1 expression (positive versus negative). | Fatigue, diarrhea, rash, and pruritus |
| 26970723 | Fehrenbacher *et al*.(2016) | NSCLC  (2^nd^ and 3^rd^ line) | 142 | Atezoluzumab 1200mg every 3 weeks | Docetaxel  75mg/m^2^  every 3 weeks | NA | Open label | Randomization 1:1, stratified by tumor-infiltrating immune cell PD-L1 expression, previous lines of chemotherapy (one vs. two), and histology (non-squamous vs. squamous). | Decreased appetite, nausea, dyspnea, and diarrhea |
| 27979383 | Rittmeyer A *et al*.(2017) | NSCLC  (2^nd^ and 3^rd^ line) | 609 | Atezoluzumab 1200mg every 3 weeks | Docetaxel  75mg/m^2^  every 3 weeks | NA | Open label | Randomization 1:1, Patients were stratified by PD-L1 expression, number of previous chemotherapy regimens (one *vs* two), and histology (non-squamous *vs* squamous). | Fatigue, decreased appetite, cough, dyspnea |
| 27718847 | Reck *et al*.(2016) | PD-L1 positive NSCLC^$^  (1^st^ line) | 154 | Pembrolizumab  200mg every 3 weeks | Platinum-based chemotherapy** | NA | Open label | Randomization 1:1, Randomization was stratified by ECOG performance-status score (0 vs. 1), tumor histologic type (squamous vs. nonsquamous), and region of enrollment (East Asia vs. non–East Asia) and did not include any provisions regarding equal distribution of enrollment across participating sites or stratification by site | Diarrhea, Fatigue, Pyrexia, and nausea |
| 27718784 | Ferris *et al*.(2016) | HNSCC  (Platinum-resistant) | 236 | Nivolumab  2mg/Kg every 2 weeks | Single agent of investigator’s choice*** | NA | Open label | Randomization 1:1, stratified by receipt of previous cetuximab therapy (yes vs. no). | Fatigue, nausea, rash, and decreased appetite |
| 25452452 | Motzer *et al 2.*(2015*)* | RCC | 167 | Nivolumab  0.3mg/Kg every 3 weeks**** | Nivolumab  2mg/Kg every 3 weeks**** | Nivolumab 10mg/Kg every 3 weeks**** |  | Randomization 1:1:1, stratified by MSKCC risk group (favorable vs. intermediate vs. poor) and number of prior treatment regimens (one vs*.* more than one) in the metastatic setting. | Fatigue, nausea, appetite decreased, diarrhea |
| 26952546 | Rosenberg *et al.*(2016) | UC  (both platinum-resistant and platinum naïve) | 310 | Atezoluzumab 1200mg every 3 weeks | NA | NA | Open label | NA | Fatigue, nausea, decreased appetite, pruritus |
| Abbreviations: Eastern Cooperative Oncology Group (ECOG), Head and neck squamous cell carcinoma (HNSCC), investigator’s chemotherapy of choice (ICC), Memorial Sloan-Kettering Cancer Center (MSKCC), Not applicable (NA), Non-small cell lung cancer (NSCLC), Not applicable (NA), Program death ligand-1 (PD-L1), Squamous cell carcinoma NSCLC (SCNSCLC), Renal cell carcinoma (RCC), urothelial carcinoma (UC).  *313 patients were excluded from meta-analysis as there were treated with both nivolumab and ipilimumab.  ** Platinum-based chemotherapy: carboplatin AUC 5-6 plus pemetrexed 500mg/m^2^ every 21 days, cisplatin 75mg/m^2^ plus pemetrexed 500mg/m^2^ every 21 days, carboplatin AUC 5-6 plus gemcitabine 1250mg/m^2^ D1 and D8 every 21 days, cisplatin 75mg/m^2^ plus gemcitabine1250mg/m^2^ D1 and D8 every 21 days, or carboplatin AUC 5-6 plus paclitaxel 200mg/m^2^ for 4 to 6 cycles.  *** Cetuximab 400mg/m^2^ once and then 250mg/m^2^ weekly, methotrexate 400mg/m^2^ weekly (may be increased to 60mg/m2), or docetaxel 30mg/m^2^ weekly (may be increased to 40mg/m^2^).  ****Number of patients evaluable for toxicity in each was summed for a total of 167 patients.  $ PD-L1 positivity was defined as a PD-L1 tumor proportion score of 50% or greater, assessed in formalin-fixed tumor samples at a central laboratory with the use of the commercially available PD-L1 IHC 22C3 pharmDx assay (Dako North America). | | | | | | | | | |
